# Supplementary material for: Nanocellulose from Cotton Waste and Its Glycidyl Methacrylate Grafting and Allylation: Synthesis, Characterization and Adsorption Properties
Source: Nanomaterials (Basel). 2021 Feb 13;11(2):476. doi: 10.3390/nano11020476 (PMC7918346; doi:10.3390/nano11020476)
Supplement: Supplementary file 1 [file nanomaterials-11-00476-s001.pdf]

Nanocellulose from cotton waste and its glycidyl methacrylate grafting and allylation. Synthesis, characterisation, and adsorption properties.

Elena Vismara[1]*, Giulia Bertolini[1], Chiara Bongio[1], Nicolò Massironi[1], Marco Zarattini[1], Daniele Nanni[2], Cesare Cosentino[3] and Giangiacomo Torri[3]

[1] Department of Chemistry, Materials and Chemical Engineering "G. Natta", Politecnico di Milano, 20131 Milano, Italy
[2] Department of Industrial Chemistry "Toso Montanari", Università di Bologna, Viale Risorgimento 4, 40136 Bologna, Italy, daniele.nanni@unibo.it
[3] Istituto di Ricerche Chimiche e Biochimiche "G. Ronzoni", 20133 Milano, Italy, torri@ronzoni.it.
* Correspondence: elena.vismara@polimi.it; dedicated to Angelo Alberti, CNR, Bologna, Italy

Supplementary information

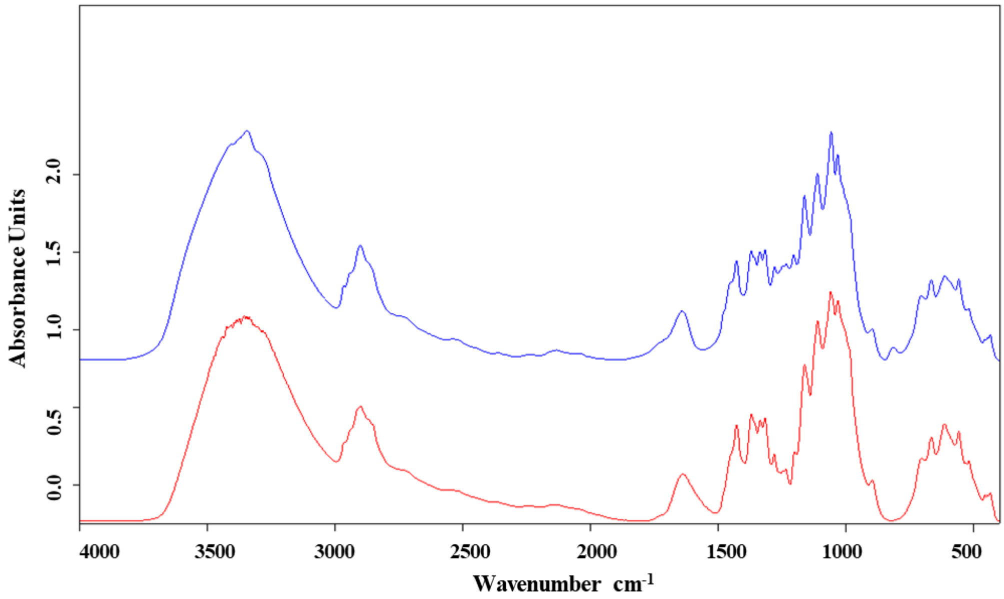

Figure S1. FT-IR spectra of HNC (blue) and CFT (red).

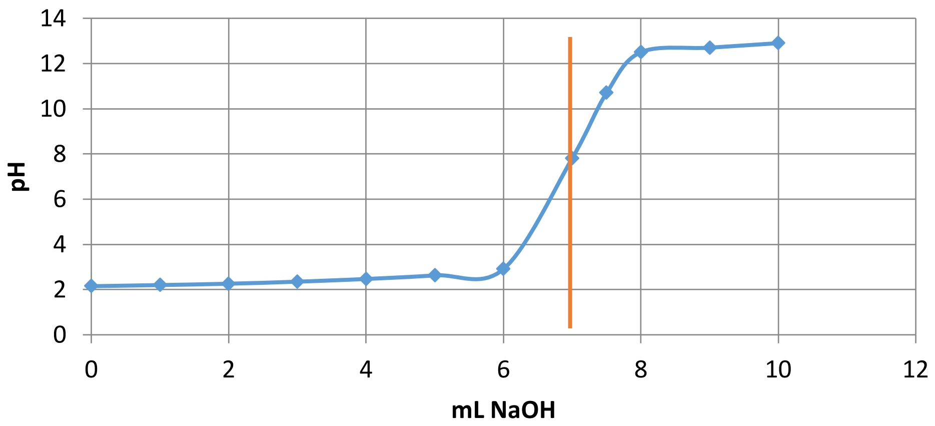

Figure S2. Graph of ONC titration

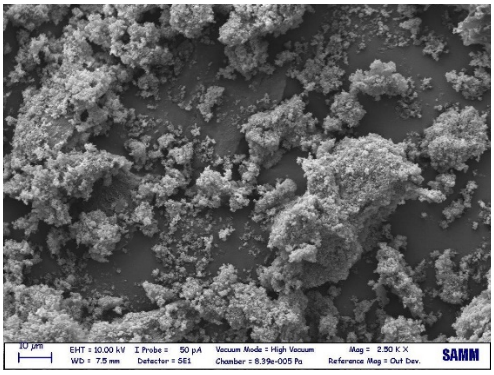

Figure S3. SEM image of ONC-GMA, MS = 2.2

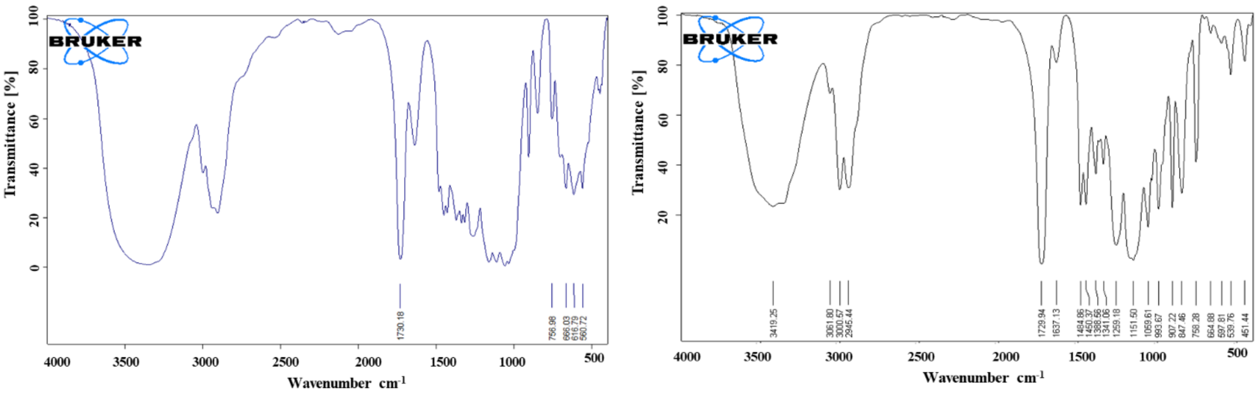

Figure S4. FT-IR HNC-GMA MS=0.43, blue (left); MS=1.5, black (right).

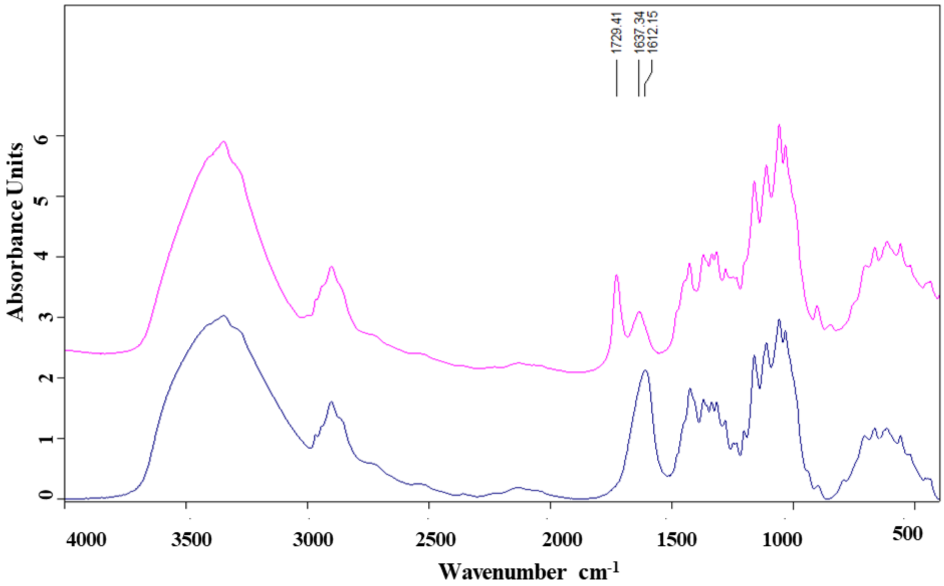

Figure S5. FT-IR spectra of salted form ONC (blue) and salted form ONC-GMA (purple).

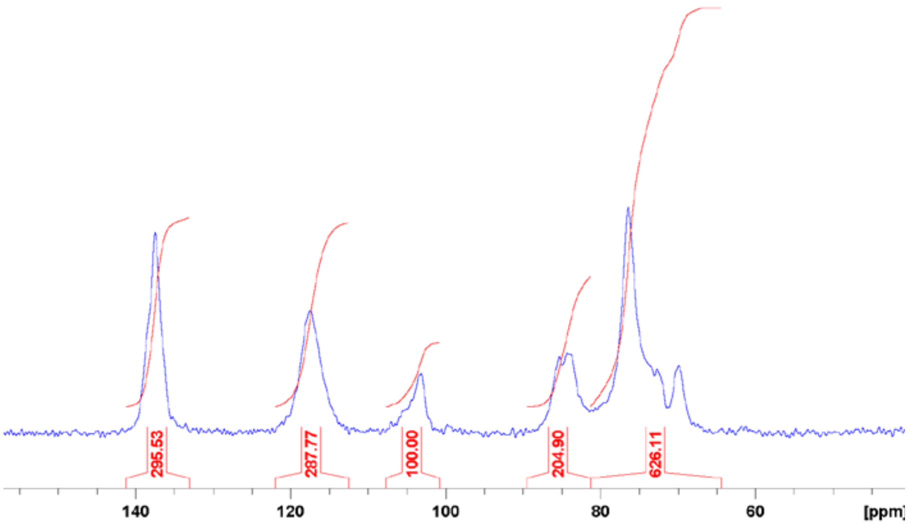

Figure S6. HNC-ALL ^{13}C CP-MAS NMR spectrum.

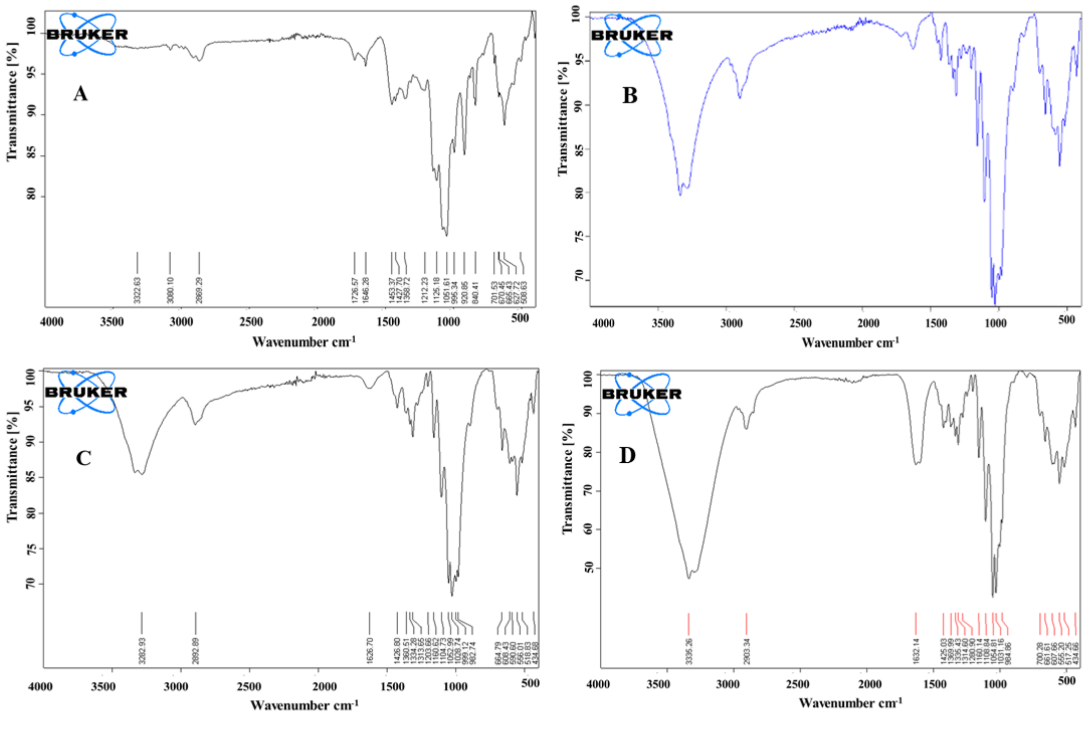

Figure S7. ATR FT-IR HNC-ALL (**A**), HNC (**B**); ONC-ALL (**C**); ONC (**D**).

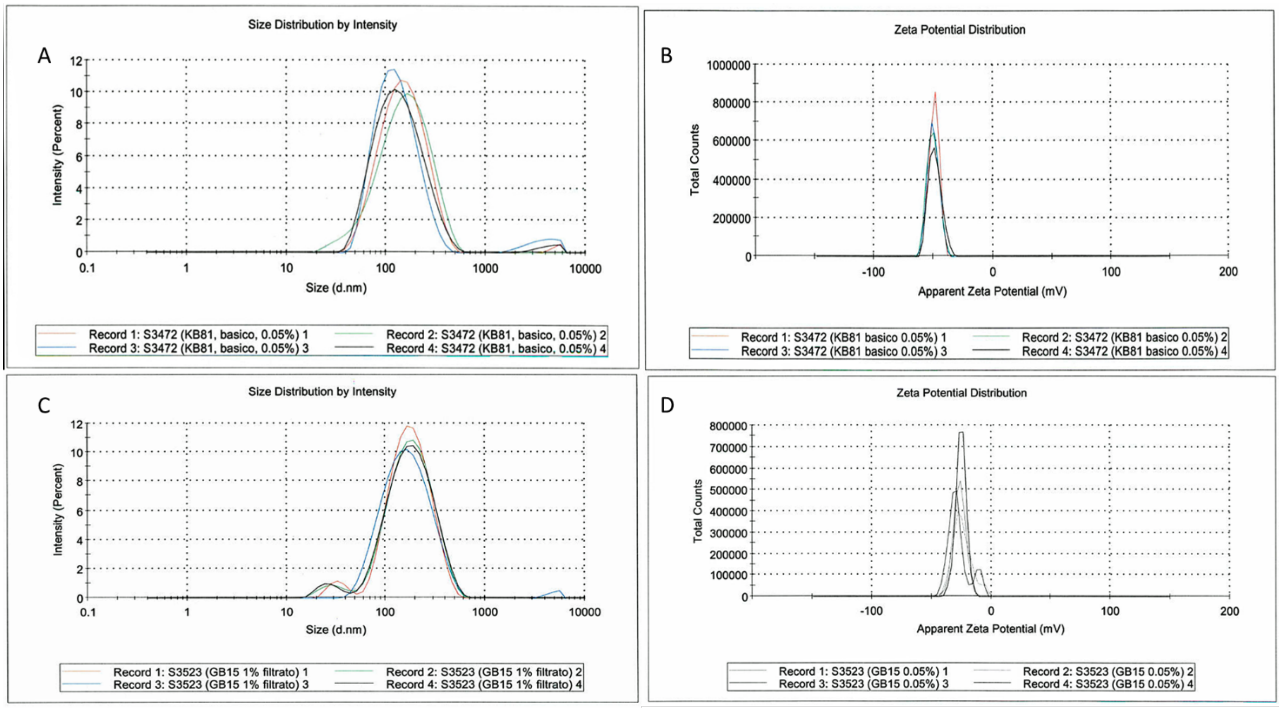

Figure S8. Hydrodynamic diameter and zeta potential (ξ) of ONC (A and B) and HNC (C and D) measured by DLS.

MolView (https://molview.org/)

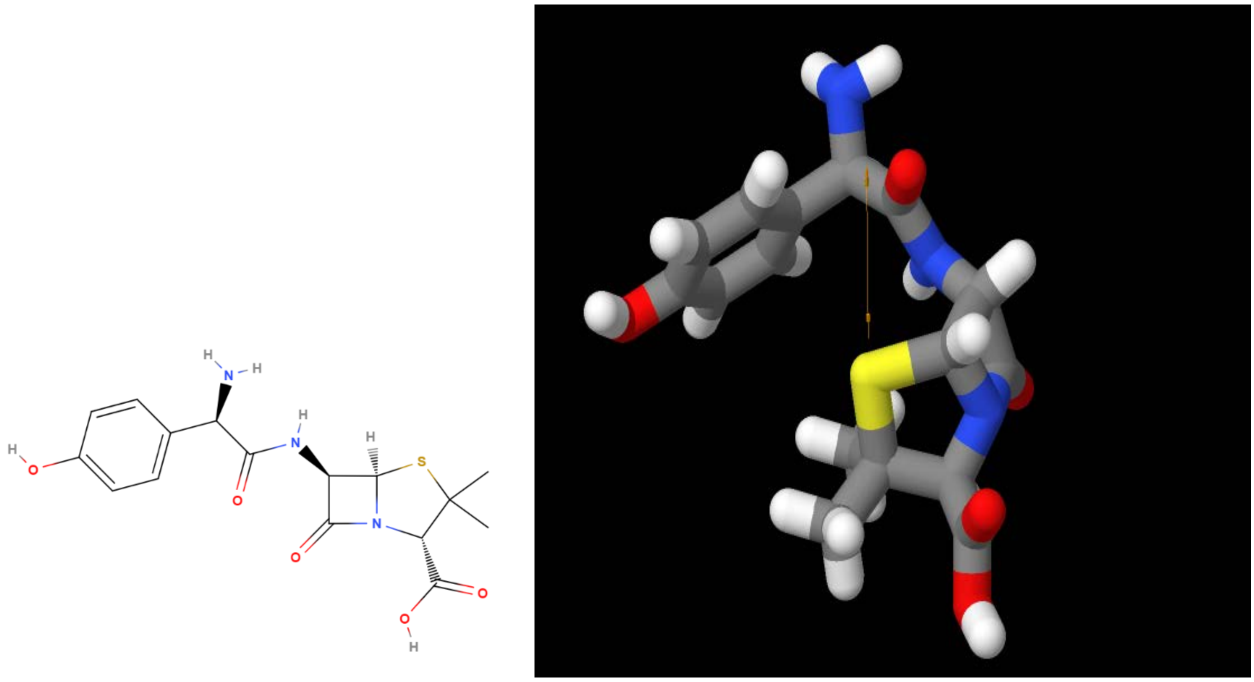

Figure S9. AM molecular structure

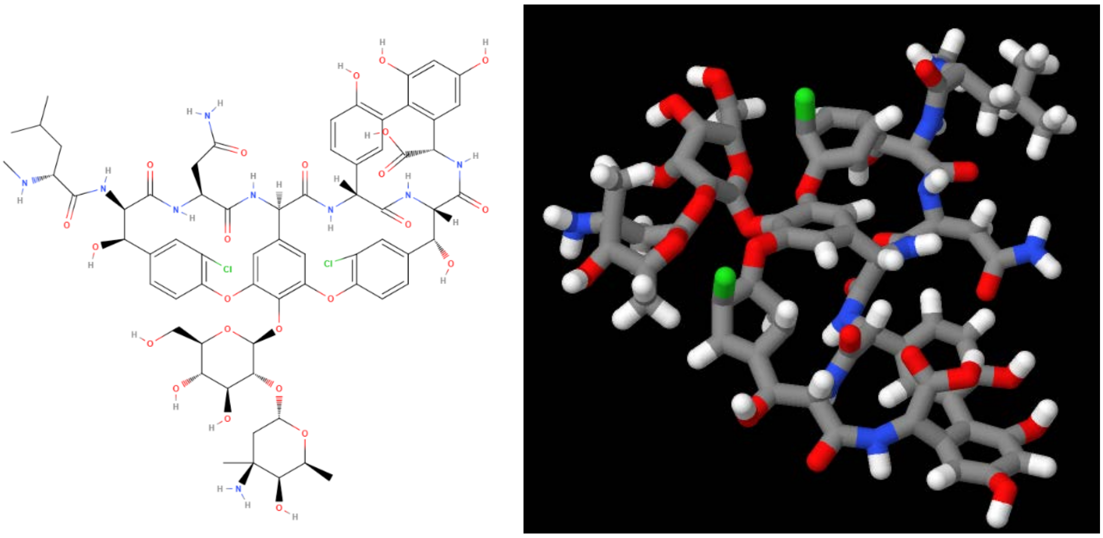

Figure S10. VC molecular structure

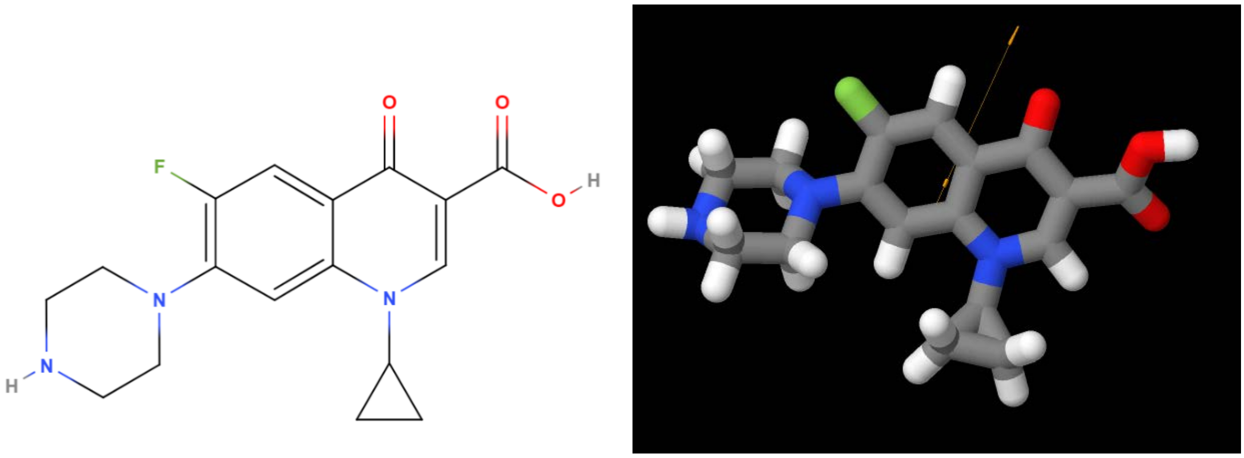

Figure S11. CP molecular structure

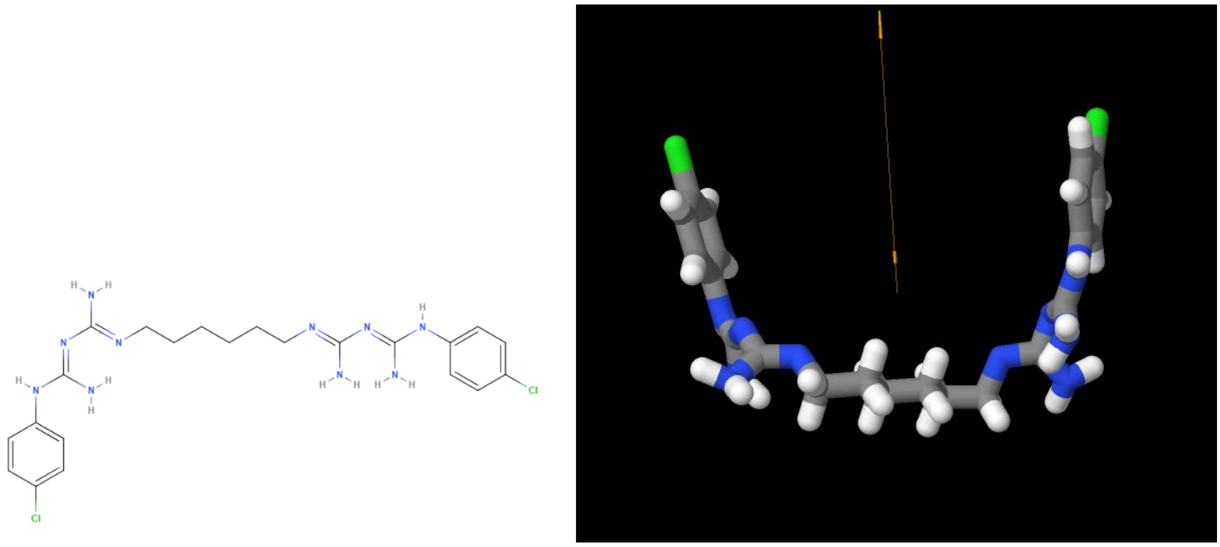

Figure S12. CHX molecular structure
